# The Use of Bioinformatics for Studying HIV Evolutionary and Epidemiological History in South America

**DOI:** 10.1155/2011/154945

**Published:** 2011-11-15

**Authors:** Gonzalo Bello, Marcelo A. Soares, Carlos G. Schrago

**Affiliations:** ^1^Laboratório de Imunologia e Aids, Instituto Oswaldo Cruz, 21040-360 Rio de Janeiro, RJ, Brazil; ^2^Instituto de Microbiologia, Universidade Federal do Rio de Janeiro, 21941-590 Rio de Janeiro, RJ, Brazil; ^3^Departamento de Genética, Universidade Federal do Rio de Janeiro, 21941-902 Rio de Janeiro, RJ, Brazil; ^4^Programa de Genética, Instituto Nacional de Câncer, 20231-050 Rio de Janeiro, RJ, Brazil

## Abstract

The South American human immunodeficiency virus type 1 (HIV-1) epidemic is driven by several subtypes (B, C, and F1) and circulating and unique recombinant forms derived from those subtypes. Those variants are heterogeneously distributed around the continent in a country-specific manner. Despite some inconsistencies mainly derived from sampling biases and analytical constrains, most of studies carried out in the area agreed in pointing out specificities in the evolutionary dynamics of the circulating HIV-1 lineages. In this paper, we covered the theoretical basis, and the application of bioinformatics methods to reconstruct the HIV spatial-temporal dynamics, unveiling relevant information to understand the origin, geographical dissemination and the current molecular scenario of the HIV epidemic in the continent, particularly in the countries of Southern Cone.

## 1. Introduction

Human immunodeficiency virus (HIV), the causative agent of AIDS, is classified into types, groups, subtypes and subsubtypes according to its genetic diversity [1]. HIV type 1 (HIV-1) is widely disseminated worldwide and can be further divided into four genetic groups: group M (*major* or *main*), group O (*outlier*), and group N (*new* or *non-M non-O*), and the most recently characterized group P [1, 2]. While HIV-1 groups N, P, and O are restricted to countries of Central Africa, notably to Cameroon, HIV-1 group M is the responsible for the AIDS pandemic, accounting for over 90% of worldwide HIV infections [3]. HIV-2 is restricted to countries of West Africa, where it also represents a minority of viral infections and is decreasing in prevalence over time [4]. Nine pure subtypes of HIV-1 group M are currently known (A–D, F–H, J, and K). Some subtypes are further divided into subsubtypes, like subtypes F (F1 and F2) and A (A1, A2, and A3). Subtypes and sub-subtypes can form additional mosaic forms though recombination of different strains inside dually or multiply infected individuals [5]. Some of these recombinant forms may further achieve epidemic relevance, giving rise to known circulating recombinant forms (CRF). To date, at least 49 CRF are recognized in diverse parts of the world (http://www.hiv.lanl.gov/content/sequence/HIV/CRFs/CRFs.html).

It is currently accepted that HIV-1M subtypes and CRF are the result of founder effects in different geographic locales, followed by localized evolution. As a consequence, such HIV-1 forms are heterogeneously spread out worldwide [6]. Subtype B, for example, is primarily found in the Americas, Western Europe, Japan, and Australia. Subtype A is typical of some sub-Saharan African countries and Eastern European countries of the former Soviet Union. Subtype C is highly prevalent in countries of sub-Saharan Africa, India, and Brazil. Some CRF may also reach relevant epidemic status, and represent the predominant strains in certain geographic regions, such as CRF01_AE in Thailand and CRF02_AG in West African countries. Indeed, it has been recently suggested that CRF02_AG is the most rapid disseminating HIV-1 variant worldwide in the last years [6].

The differential distribution of HIV-1 subtypes and CRF also impact on their worldwide prevalence estimates. For instance, while subtype B dominates in several developed countries with the lowest HIV prevalence rates, it accounts for only 11% of the worldwide infections. Conversely, subtype C accounts for nearly half of worldwide infections, as it prevails in countries with the highest HIV infection rates such as South Africa, and Botswana, or in highly populated countries like India [6].

## 2. HIV-1 Diversity in South America

South America follows the HIV molecular epidemiology commonly seen in the Americas, with HIV-1B being the most prevalent. However, a number of regional specificities are also observed. Brazil, the largest country of the continent, and which accounts for roughly two thirds of the infections, has likely the highest reported diversity. In addition to HIV-1B, other subtypes such as F1, C, and a number of B/F and B/C recombinants cocirculate [7–11]. In other South American countries, HIV-1B has been mostly reported, with the exception of Argentina and Uruguay, where a large number of B/F recombinants circulate at high proportion [12–16].

As HIV-1B, C, and F1 are the predominating pure subtypes in the area, a profusion of CRF comprising those subtypes have been characterized in South America. Those included the B/F-derived CRF12 in Argentina, Bolivia, Chile, Paraguay and Uruguay [17–19], CRF17 in Argentina and Paraguay [17, 19], CRF28, CRF29, CRF39, CRF40, and CRF46 in Brazil [20–22], CRF38 in Uruguay [15], CRF44 in Chile [23], and the B/C-derived CRF31 in southern Brazil [24]. All these CRF are thought to have been generated locally by recombination of the prevailing HIV-1 subtypes. Other HIV-1 clades have also been sporadically detected in South America, such as the subtype D and the CRF02_AG in Brazil [25–27] and the CFR16_AD and CRF06_cpx in Argentina [14, 28]. These forms were likely introduced by African immigrants, and have not achieved epidemic relevance to date.

## 3. Theoretical Basis of Evolutionary Bioinformatics Methods Applied on the Study of HIV

### 3.1. Phylogenetics

The most frequent evolutionary analysis performed on HIV sequences is usual phylogenetic inference. Phylogenetic trees provide essential information on the structure of the genetic diversity of the lineages. As in common hierarchical cluster analysis, phylogenies depict major groups in the data. However, such groups are related in time via the vertical process of genetic information passage, and, thus, tree topologies also depict the evolutionary history of the sequences. Phylogenies have been central to understand HIV evolution. The categorization of the major groups, subtypes, and their relationships were attained by phylogenetic inference [29]. Also, the characterization of HIV as a zoonosis and the identification of its geographic origins were permitted, because tree topologies for primate lentiviruses were known [30, 31].

Contemporaneously, phylogenetic tree reconstruction is accomplished by two different statistical approaches, maximum likelihood (ML) and Bayesian inference (BI). Both methods explicitly use Markov models of sequence evolution, and it is difficult to assert which one has superior performance [32]. ML inference would ideally address the phylogenetic problem by finding the tree that maximizes the likelihood function*f*(*D* | *T*), where *D* is the sequence alignment. In practice, however, such function is nonexistent because each topology has its own likelihood function *f*(*D* | *θ*), where *θ* is the vector of evolutionary parameters associated with a specific tree [33]. This peculiarity seems not to be an issue, since ML methods perform very well in simulations [34]. The Bayesian approach deals with the function *f*(*T* | *D*), the posterior probability of the tree, which is fundamentally the product between the tree likelihood and the tree prior

(1) $$f\left(T\;|\;D\right)=\frac{f\left(D\;|\;T\right)f\left(T\right)}{\sum_{i=1}^{S}f\left(D\;|\;T_{i}\right)f\left(T_{i}\right)}.$$

The denominator of the Bayes formula is the normalizing term. It is the sum of products between likelihood and prior of all *S* tree topologies. Thus, the posterior probability of a given tree will lie between 0 and 1. This function is impossible to study analytically because the number of tree topologies conceivable is generally astronomical. Therefore, the posterior density is obtained via the Markov chain Monte Carlo (MCMC) technique [35].

Both ML and BI methods are computationally very intensive, but ML have become faster with recent developments of powerful heuristic search algorithms like PhyML [36], RAxML [37] and Garli [38], to cite a few. This has enabled the analysis of large data sets and the assessment of clade support via bootstrap [39]. BI is mainly implemented in the software MrBayes [40], which provides sophisticated models of sequence evolution and data handling. The use of MCMC algorithms, such as Metropolis-Hastings, bestows BI the capacity to adopt more complex models of sequence evolution in order to capture the biological reality of the evolutionary process [41].

In practice, if the researcher is interested in unveiling general patterns of genetic and spatial structuring of the viral diversity, phylogenetic inference should be performed on orthologous nonrecombining genome regions. This may be difficult to know *a priori*. Fortunately, there are analytical tools designed to identify recombination breakpoints on sequence alignments. For HIV, the SimPlot software [42] has been widely used. It implements the bootscanning, a simple sliding window strategy along the alignment in search of regions with conflicting phylogenetic signals [43]. Such regions will group with different reference sequences with significant bootstrap support. Although very useful and intuitive, this analysis does not offer a standard statistical testing framework. In this case, other methods have been recently developed. For instance, Pond and collaborators [44] have described a genetic algorithm to detect recombination breakpoints, implemented in GARD software (http://www.datamonkey.org/GARD/). This method uses the Akaike information criterion to choose among several breakpoints conformations.

### 3.2. Phylogeography

When studying the structure of the genetic diversity, it is often evident that lineages have a nonrandom distribution in space. In HIV epidemics, there are several examples of virus lineages of monophyletic origin with restricted spatial distribution [45]. When such a pattern is found, it means that the entrance of virus in the region was a unique event and it is possible to track its geographic origin by verifying the sister-group relationships on the phylogeny. Instead, if HIV sequences collected in an area are not monophyletic, we may still be able to track the geographic origins of the several independent virus lineages [46–49]. Actually, assessment of the spatial structure of the genetic diversity offers a relevant measure of the spatial dynamics of the epidemics that are critical to design public health policies [50].

Phylogeographic analysis has been gaining much attention from population biologists [51–53], since the spatial dynamics of organisms offer important insights into biological process from speciation to dispersion rates. Potentially applied to HIV research, a Bayesian implementation of ancestral reconstruction of spatial distribution was proposed by Lemey et al. [54], who implemented a Markov modeling of the discrete state of space of geographic localities, where the most parsimonious path is chosen by Bayesian stochastic search variable selection. This algorithm was subsequently extended to incorporate diffusion on a continuous space [55].

### 3.3. Timescale

Rooted phylogenetic trees impose a chronological direction on phylogenies. When rooted trees are ultrametric, branch lengths are proportional to the time elapsed since the separation of lineages. However, in the absence of external information on absolute times, chronologies can only be measured in units of mutations per site. If a calibration point is known, the branch lengths can be measured in absolute time (years, months, etc.). It is possible to extrapolate this information to the entire tree if evolutionary rates are homogeneous among lineages. This is the strict molecular clock [56]. In rapidly evolving pathogens, such as HIV, it is also possible to estimate divergence times by knowing the age of the leaves of the tree. This strategy is called tip dating and can be applied on heterochronous data sets, that is, when a significant number of mutations between sampling years occurs that enable the direct inference of the absolute mutation rate [57]. Populations that present such features are said to be measurably evolving [58].

As expected, the strict molecular clock rarely holds and, thus, a family of methods that estimates divergence times by relaxing the rate homogeneity assumption was developed over the last decade [59–63]. Although there are significant differences among these methods, they all share the same fundamental aim: the decomposition of branch lengths into absolute times and rates [64]. As in phylogenetic inference *per se*, ML and BI have been applied to tackle this problem. In an ML framework, branch lengths are decomposed by using multiple local molecular clocks [63, 65] or via rate-smoothing functions [66]. It is the Bayesian estimation of divergence time, however, that has gained much attention since its original proposition [35, 67]. This is mainly because the BI allows the usage of sophisticated models of evolutionary rate evolution, such as correlated [62] and uncorrelated models [59]. Moreover, calibration information can be flexibly incorporated by the adoption of probability distribution as priors [59].

In HIV research, ML and BI approaches have been widely applied in association with tip dating to estimate evolutionary rates and divergence times, both within and among hosts [68–71].

### 3.4. Demography

Another recent technical development in the study of HIV evolution is the application of methods derived from the coalescent theory. In a now classic paper, Kingman [72] derived the properties of genealogies obtained when population genetics is considered backwards in time. When doing this, several relevant parameters might be estimated, such as the time of the most recent common ancestor of alleles. The theoretical framework of the coalescent was extended to DNA sequences [73] and has also been applied to the study of evolutionary demography [74–76]. When the effective population size changes, the topology of the gene genealogy is expected to change in a predictable manner. Thus, demographic parameters like the growth rate might be inferred from the tree topology and different demographic models can be formally tested in a likelihood framework [75, 77]. For instance, it is possible to explicitly test if the likelihood of the data under the logistic growth model is significantly greater than the likelihood under the simple exponential growth or constant population size.

In a Bayesian framework, one may estimate the posterior probability of a demographic model given the data, *f*(Θ | *D*). Demographic models are incorporated via the coalescent prior function *f*(*g* | Θ), which computes the likelihood of a given genealogical tree topology *g* given the demographic model Θ. Evidently, the model of sequence evolution *ψ* should also be considered, resulting in the following posterior distribution:

(2) $$f\left(\Theta ,\psi ,g\;|\;D\right)=\frac{1}{Z}f\left(D\;|\;\psi ,g\right)f\left(g\;|\;\Theta \right)f\left(\Theta \right)f\left(\psi \right),$$

where *Z* is the denominator of the Bayes formula and *f*(Θ) and *f*(*ψ*) are the demographic and sequence evolution model prior functions respectively. Thus, the marginal density *f*(Θ | *D*) is estimated by averaging over all *ψ* and *g* values

(3) $$f\left(\Theta \;|\;D\right)=\frac{1}{Z}\underset{g}{\sum }\overset{}{\underset{\psi }{\int }}f\left(D\;|\;\psi ,g\right)f\left(g\;|\;\Theta \right)f\left(\Theta \right)f\left(\psi \right)d\psi .$$

From the above equation, it is evident that, in a Bayesian framework, tree topologies (*g*) are considered a nuisance parameter, since Θ is integrated over the topological space [78]. 

The demographic dynamics of HIV populations are, however, much more complex than the simplistic assumptions made by common growth models. Besides that, it is difficult to know *a priori *which model is appropriate to describe the demographic history. A family of coalescent techniques known as “skyline plots” was developed with the purpose of extracting demographic information from gene genealogies without assuming an explicit model. Skyline plots depicts the variation of the effective population size through time by the adoption of a piecewise demographic mathematical description of the data [75]. As initially proposed, the method acts on a fixed ultrametric gene genealogy of *n* terminals. For each *i*th interval between nodes, that is, coalescent events (Δ*u*), the fundamental quantitative relation between the number of lineages (*k*) and effective population size (*θ*) is applied via the function

(4) $$\log f\left(g\;|\;\Theta ,A\right)=\overset{n-1}{\underset{i=1}{\sum }}\log\frac{k_{i}\left(k_{i}-1\right)}{{2\theta }_{i}}-\frac{k_{i}\left(k_{i}-1\right)\Delta u_{i}}{{2\theta }_{i}},$$

where Θ is the vector of effective population sizes and *A* is number of classes used to group the *i* intervals [74]. In the classic skyline plot, *A* = *i*. This equation describes some intuitive population genetics principles. For instance, if the effective population size decreases, the time interval between coalescent events will become shorter as the number of lineages increases.

Although this approach is much more realistic than the adoption of a specific model of population size change, there are still some drawbacks. Firstly, since the method acts on ultrametric trees, the strict molecular clock must be assumed. Also, the gene genealogy *g* must be fixed, and hence, it is considered known without error. This is a twofold problem, because it is obvious that phylogenetic inference is subject to errors, and, frequently, the researcher is only interested in demographic parameters instead of topological relationships of the sequences. The proposition of the Bayesian skyline down sized these problems [74]. In a Bayesian context, gene genealogies *g* may be considered a nuisance parameter and demographic parameters can be estimated by integrating over the topological space. This is achieved via MCMC algorithms [78]. The Bayesian skyline method needs *a priori* determination of the number *A* of intervals between coalescent events. This is subjective and largely depends on the historical information content of the alignment. Another weakness is that effective population sizes are assumed to be correlated in successive coalescent intervals. To surpass this issue, the Bayesian skyride, a method that penalizes the change of this parameter between intervals was developed [79].

Piecewise demographic models are recommended to be applied when sequence alignments bear significant demographic information [80]. In practice, this is difficult to determine, but studies involving measurably evolving populations, like the majority of HIV datasets, are suitable for such analysis. Ideally, the power of the skyline methods is increased when multiple *loci* are used. In order to incorporate the information from multiple *loci*, Heled and Drummond [81] have proposed the extended skyline method. However, to gain statistical power, multiple unlinked nonrecombining *loci* must be used. Unfortunately, this is not feasible for HIV datasets and researchers may only try to reduce stochastic error of the analysis by augmenting the number of nucleotide sites. For the moment, none of these methods have incorporated population structure in their framework. Since it is not clear how spatial population structure affects skyline plots, when such information is known *a priori*, it is better to investigate each population separately.

Finally, the coalescent theory used in demographic estimation measures time in generations. Therefore, when calibrating a gene genealogy, in which branch lengths are measured as the number of mutations per site, one should ideally enter chronological information in generations (*τ*). When this is the case, skyline plots will depict the variation of the absolute effective population size (*N*_*e*_) through the generations. Most commonly though, in HIV studies, chronological information is measured in years via tip dating. Thus, the unit of the *y*-axis of the skyline plots is the product *N*_*e*_ × *τ*, where *τ* is the generation time in years.

Demographic inference using the methods described above is basically implemented on the BEAST software [80]. In practice, researchers will use the skyline model as topological prior while simultaneously inferring divergence times and evolutionary rates in a relaxed or strict clock framework. Therefore, in a single analysis, population demography, the time of the most recent common ancestor of lineages and evolutionary rates are coestimated considering tree topology as a nuisance parameter, since values are averaged over the topologies sampled during the MCMC run. Actually, these tree topologies might be summarized to graphically represent historical process that generated the sequences.

### 3.5. Origin and Timescale of HIV-1 Clades in South America

Several studies have been performed to reconstruct the origin and timescale of major HIV-1 clades circulating in South America, including subtypes B, C, F1, and several CRF lineages.

Subtype B viruses circulating in South America belong to the “pandemic” clade that migrated out of Haiti around 1969 (1966–1972) and spread through the world [82]. In most of the South American countries, HIV-1B epidemic probably resulted from introduction of multiple strains and subsequently spread within local networks although this hypothesis has not been formally tested. Some country-specific subtype B polymorphisms, however, have been described in South America. While most (~95%) subtype B viruses of the pandemic clade carry a GPGR motif at the tip of the V3 loop, the Brazilian subtype B epidemic is characterized by roughly similar proportions of strains containing the common GPGR motif and the unusual GWGR motif [9, 83–86]. Phylogenetic analyses of Brazilian subtype B *env* sequences showed that GWGR isolates formed a monophyletic cluster (B-Br clade) nested within the basal GPGR Brazilian sequences [87, 88], supporting the hypothesis that GWGR strains originated from a single founder GPGR Brazilian strain. The TMRCA of the B-Br clade was estimated to be 1966 (1954–1975) [87], which roughly coincides with the age of the subtype B pandemic clade, suggesting that the founder event that originates the Br-B lineage probably occurred at the beginning of the subtype B epidemic in Brazil. This hypothesis is supported by a recent analysis of HIV-positive serum samples collected in Brazil in 1983 that confirms the circulation of GWGR isolates at such very early stage of the Brazilian epidemic [89]. Recent phylogenetic studies using full-length subtype B genomes showed that GWGR viruses are evenly dispersed among GPGR Brazilian strains [84, 89], suggesting a polyphyletic origin of GWGR strains. Such observation, however, could result from extensive intrasubtype recombination between GWGR and GPGR variants, rather than from independent evolution of multiple GPGR strains into GWGR strains.

The circulation of “pure” subtype F1 viruses in South America seems to be almost restricted to Brazil. Despite the high prevalence of BF1 recombinants in countries from the Southern cone (Argentina, Brazil, Bolivia, Chile, Paraguay, and Uruguay), full-length subtype F1 viruses, or even subtype F1 *pol* sequences, are very rarely found outside Brazil [13–16, 18, 90]. Phylogenetic analyses of full-length and partial genome subtype F1 sequences consistently showed that Brazilian and South American viruses form a monophyletic group when compared to subtype F1 viruses from other countries around the world [87, 90–93]. Within such South American lineage, subtype F1 *env* fragments of BF1 recombinants from Argentina, Bolivia, Chile, Paraguay, and Uruguay form a monophyletic cluster nested within the basal subtype F1 Brazilian sequences [90]. These results indicate that South American HIV-1F1 and HIV-1BF1 epidemics are the result of the introduction of a single founder subtype F1 strain through Brazil, followed by expansion and recombination of this virus with local subtype B viruses. Recent evidence suggests that this founder strain came from the Democratic Republic of Congo (DRC), as it does not resemble other HIV-1F1 worldwide lineages such as those found in Angola and Romania [91]. Three independent studies based on the analysis of *env* and *pol* sequences date back the TMRCA of the Brazilian and South American subtype F1 clade to between the middle 1970s and the early 1980s: 1976 (1966–1982) [90], 1978 (1972–1983) [87], and 1980 (1975–1985) [93]. One study based on the analysis of *gag* sequences, however, traced the TMRCA of South American subtype F1 clade back to 1969 (1959–1978) [92], similar to the origin of the subtype B pandemic clade.

The pervasive recombination between subtype B and F1 viruses in South America created a large variety of intersubtype BF1 recombinants, some of which have disseminated across several individuals, gaining the status of CRFs_BF. The most widespread of these CRFs is the CRF12_BF that circulates in Argentina, Uruguay, Bolivia, Chile, and Paraguay. Epidemiological data revealed that CRF12-like BF1 viruses have been circulating in Argentina since the mid 1980s [94], but the exact origin of this recombinant clade is still uncertain. Three studies have reconstructed the timescale of the CRF12_BF using Bayesian relaxed-clock methods, with quite different results. The first study, based on the analysis of *vpu* CRF12-like sequences from Argentine children, estimated the TMRCA of this clade at 1992 (1981–1996) [95]. The second study, based on the analysis of a large data set of *pol *CRF12-like sequences from Argentina and Uruguay, dated back the origin of this clade to 1983 (1978–1988) [96]. The third study, based on the analysis of subtype B *pol* gene fragments from CRF12_BF viruses from Argentina, suggests that the origin of this CRF could be dated back to 1969 although the confidence interval (CI) of such estimate was extremely large (1946–1981) [92]. The timescale of other CRF_BF viruses with more restricted circulation have been also estimated. The TMRCA of the CRF38_BF clade that circulates in Uruguay was traced to 1986 (1981–1990) [96], while the CRF28_BF and CRF29_BF clades circulating in the southeastern region of Brazil probably evolved from a common BF1 recombinant ancestor that existed around 1989 (1987–1993) [97]. Thus, the South American CRFs_BF represent “old” viral lineages probably generated during the 1980s, shortly after the introduction of subtype F1 into the region.

**Figure 1:**
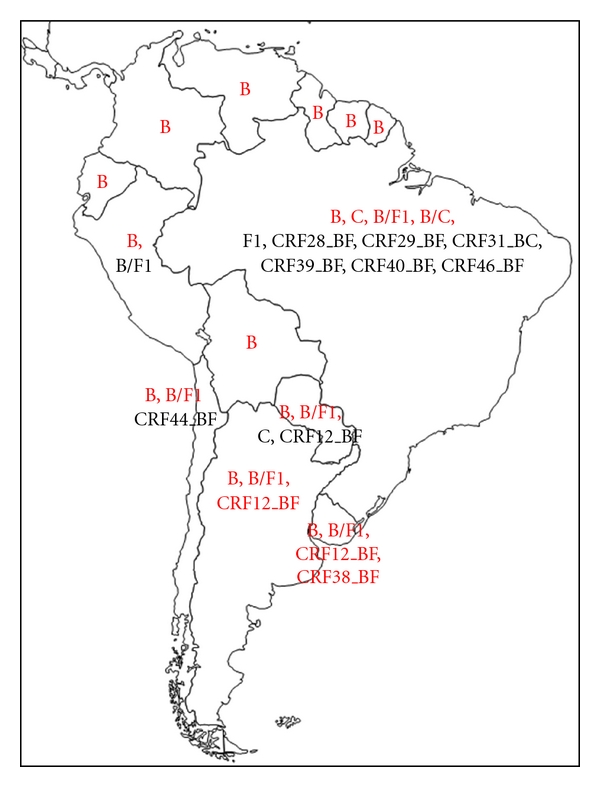
Political map of South America depicting the major HIV-1 variants occurring in each country. Major variants (prevalence above 5%) are listed in red.

The circulation of HIV-1C in South America is mainly concentrated in the southern region of Brazil. Most studies performed to date support the notion that South American HIV-1C epidemic was also the result of a single founder event followed by local dissemination of the new virus and suggested the entrance spot at southern Brazil [11, 98–101]. Although one study claimed a possible entrance of HIV-1C through Argentina [102], epidemiological data are not consonant with this hypothesis. Several studies have been performed to trace the origin of HIV-1C lineage that colonized the region. Some studies traced the origin of such clade to somewhere in East Africa, most likely to Burundi, Kenya, or Ethiopia [98, 99]. Others proposed the introduction of HIV-1C in southern Brazil from Mozambique by means of Portuguese colonization of the latter country and migration to Brazil [103], but phylogenetic and molecular evidence did not support that proposal [104]. An additional study suggests that HIV-1C migrated from East Africa to Brazil through a network of men who have sex with men (MSM) from London, England [100]. More detailed analyses and additional samples are, however, required to fully elucidate such relationships and the actual migration history of subtype C to Brazil. It is also unclear when the HIV-1C founder strain was introduced into the Brazilian population. The first study to estimate the timescale of Brazilian subtype C lineage used an ML strict-clock approach and points the origin of such clade to the early 1990s [105]. A second study employed a Bayesian strict-clock method and estimates the onset date of the Brazilian subtype C epidemic at around 1987, but the CI obtained was very wide (1956–1998) [106]. More recent studies based on Bayesian relaxed-molecular clock models described older mean TMRCA estimates. Two independent studies indicate that the TMRCA of Brazilian subtype C clade dates back to the early 1980s: 1980 (1972–1987) [100] and 1982 (1972–1988) [98]. Another study suggests that subtype C introduction in Brazil could be even older, dating back to between 1960 and 1970, although important variations were observed in such study across distinct viral genomic regions analyzed: gp41 (1962; 1950–1972), RT (1968; 1959–1976), and p24 (1977; 1966–1986) (Figure 1) [101].

The cocirculation of HIV-1 subtypes B and C in the southern Brazilian region also creates a variety of intersubtype BC recombinants, including one CRF designated as CRF31_BC, which is particularly prevalent in Rio Grande do Sul, the southernmost state of Brazil. Phylogenetic and informative site analyses comparing the CRF31_BC lineage with Brazilian subtypes B and C clades clearly supports a local origin of this recombinant form [24, 107]. Two independent studies have employed Bayesian clock methods to estimate the timescale of the CRF31_BC epidemic using the recombinant *pol* (PR/RT) gene fragment. Both studies estimated the TMRCA of the CRF31_BC clade at around the late 1980s: 1987 (1967–1998) [106] and 1988 (1979–1993) [107]. The identification of CRF31_BC-infected Brazilian individuals with HIV diagnosis as early as 1990 [106, 107] is fully consistent with the estimated origin of this clade during the 1980s. Although CRF31_BC viruses certainly derived from a single BC recombinant ancestor, phylogenetic analyses of subtype C genomic regions (integrase, *env*-gp120 and *env*-gp41) from CRF31_BC viruses reveal that those viruses do not formed a monophyletic cluster within subtype C Brazilian clade, but were evenly dispersed among subtype C Brazilian viruses [107]. This observation resembles the lack of monophyletic clustering of subtype B GWGR variants outside the *env* region and could be also explained by the widespread recombination between CRF31_BC and subtype C Brazilian strains.

The different studies performed up to date support two opposite scenarios for the timescale of the HIV-1 epidemic in South America. Some studies suggest that HIV-1B was the first to colonize the continent between the middle 1960s and the early 1970s, followed by HIV-1F1 and HIV-1C some years later (between the middle 1970s and the early 1980s). Other studies, however, supports the concurrent introduction of all three HIV-1 subtypes in South American between the middle 1960s and the early 1970s. Determine the actual timescale of the major HIV-1 clades circulating in South America is of paramount importance for understanding the circumstances surrounding the emergence of such epidemics and their subsequent dissemination dynamics.

The great variation in the mean estimated TMRCA of some South American clades: subtype C (1962 to 1990), subtype F1 (1969 to 1980), and CRF12_BF (1969 to 1992), as well as the wide range of the CI of many estimates (up to 30 years) exposes the important challenge to derive reliable timescales for HIV evolution. The considerable rate variation among HIV-1 lineages at the population level produces a departure from the clock-like evolution that can seriously hamper our ability to accurately estimate the evolutionary rate and the TMRCA of HIV-1 [108]. However, uncertainty in TMRCA estimates of South American HIV clades remains despite the implementation of more realistic and phylogenetically accurate “relaxed” molecular clocks models that accommodate such rate variation among lineages [59]. It is possible that variation in the size and the nature of data sets [109], and/or time intervals at which sequences are sampled [110] may also disturb the substitution rates and divergence date estimations.

### 3.6. Demographic History and Epidemic Potential of HIV-1 Clades in South America

The development of phylogenetic approaches that incorporate the coalescent theory of population genetics enabled us to infer the demographic history and epidemic potential of major South American HIV-1 clades.

The first study published in 2005 used an ML coalescent-based approach to explore the demographic history and epidemic potential of HIV-1 subtypes B and C in Brazil, based on the analysis of *pol* sequences collected up to 2001 [105]. That study suggested that both HIV-1 subtypes were spreading exponentially in Brazil and that the mean subtype C growth rate (0.6–0.8 year^−1^) was about twice that of subtype B (0.2–0.4 year^−1^) (Table 1). These observations were confirmed by a second study that used a Bayesian coalescent-based approach to estimate the growth rate of Brazilian subtypes B and C epidemics under a demographic model of exponential growth (Table 1) [106]. Thus, initial studies supported the existence of a growing HIV-1 epidemic in Brazil by the 2000s and suggest that subtype C was spreading at a significantly faster rate than subtype B.

Subsequent studies, however, pointed to a different epidemic scenario. Bayesian skyline plot analyses of HIV-1 *env* and *pol* sequences collected from Brazilian patients up to 2005–2006 indicated that subtype B and F1 epidemics in the southeastern region and subtype C epidemic in the southern region were better explained by a model of logistic growth, characterized by an initial period of rapid exponential expansion followed by a decline in growth rate since 1985–1995 [87, 111]. Such proposed slowdown of the growth rate of HIV-1 subtypes B, F1 and C epidemics coincides with epidemiological information that reveals that after a period of explosive growth during the 1980s and 1990s, the number of new AIDS cases annually reported in the southeastern and southern Brazilian regions has shown a trend toward stability since 1995 and 2000, respectively [112].

**Figure 2:**
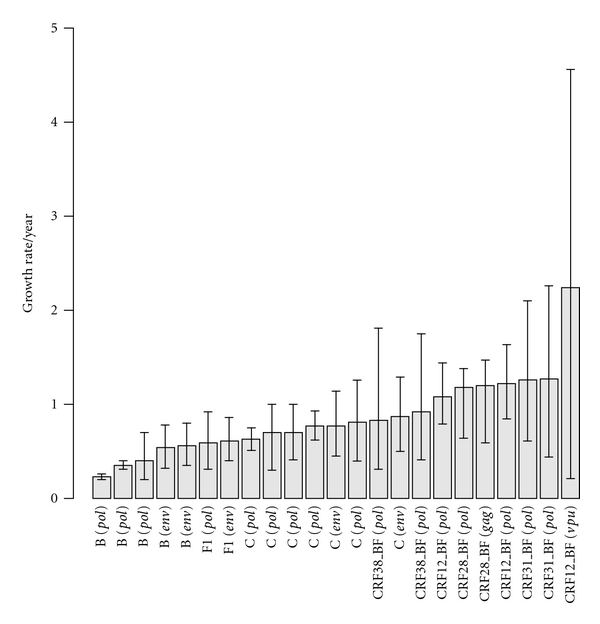
Histogram depicting the variance in the epidemic growth rates of HIV-1 clades in South America. Refer to Table 1 for further details regarding applied models and references describing estimates.

The mean growth rate of Brazilian subtype B epidemic estimated for the model of logistic growth (0.45–0.55 year^−1^) [87] was significantly higher than that previously estimated for the model of exponential growth [105], yet lower than that described for the North American subtype B epidemic (0.8 year^−1^) under the same logistic demographic pattern [113]. By contrast, the mean estimated growth rate of subtype C epidemic for the model of logistic growth (0.7–0.9 year^−1^) [111] was similar to that previously obtained for the exponential one [105]. The mean initial growth rate of subtype F1 epidemic (~0.6 year^−1^) was in-between those estimated for subtype B and C [87]. Although the mean growth rates for the logistic growth model support the notion that subtype C clade exhibited an initial rate of spread slightly higher than subtypes B and F1, the CI intervals of such estimates displayed a great overlap (Table 1 and Figure 2). Thus, it is unclear whether the initial rate of dissemination of different Brazilian HIV-1 subtypes was or not significantly different.

Some studies have also used the Bayesian skyline coalescent-based method to reconstruct the epidemic history of major South American CRFs clades. All studies indicate that the effective number of infections by CRF_BF (12, 28/29, and 38) and CRF31_BC experienced a fast exponential growth over a 5–15 year period after their emergence, but then decreased toward the present following the same logistic model of population growth described for parental HIV-1 subtypes [95–97, 111]. An initial study of the CRF12_BF epidemic in Argentine children indicated an extremely rapid rate of population expansion for this clade (2.2 year^−1^), but the CI of such estimate was huge (0.21–4.56 year^−1^) [95]. A more precise estimate was recently obtained through the analysis of CRF12_BF viruses circulating in Argentina and Uruguay [96]. According to this study, the CRF12_BF epidemic spread in those countries with an initial mean growth rate of around 1.2 year^−1^ (0.85–1.6 year^−1^), which is about half of that previously obtained for this CRF, but still higher than those reported for Brazilian HIV-1 subtypes B, F1, and C. Similarly, high initial mean growth rates were also recently estimated for the CRF38_BF clade in Uruguay (0.9 year^−1^) [96], and for the CRF28/29_BF (1.2 year^−1^) [97] and CRF31_BC (1.3 year^−1^) [111] clades in Brazil (Table 1).

Thus, Bayesian coalescent-based analyses performed to date suggest that major HIV-1 clades circulating in South America followed the same overall demographic pattern described for HIV-1 subtype B in USA and some European countries [46, 113–116], characterized by an initial phase of rapid expansion followed by a recent period of stabilization. Such a recent decline in the growth rate of these HIV-1 epidemics may be the consequence of implementation of efficient prevention campaigns after the official recognition of HIV/AIDS in the early 1980s, and/or the result of a saturation of high-risk transmission networks in concentrated HIV/AIDS epidemics. One important limitation of the studies performed in Brazil, however, is that most HIV-1 samples were derived from the major metropolitan areas of the southern and southeastern regions, and may not represent the demographic trend of HIV epidemics in other localities. It has been documented that while the number of new AIDS cases has remained stable over the last years in the cities with >500,000 inhabitants from the southern, southeastern, and central-west regions, that number continuous to growth in the northern and northeastern regions as well as in the cities with <50,000 inhabitants from all over the country [112].

Despite some overlap of the CI of growth rates estimates (Table 1), Bayesian coalescent-based analyses also suggest that CRFs have spread at a rate much higher than parental HIV-1 subtypes in the South American population (Figure 2). A very attractive hypothesis to explain such observation is to propose that CRFs display a higher transmissibility than their parental HIV-1 subtypes. Indeed, such scenario has been suggested to explain the predominance of CRF02_AG in West Central Africa [117]. According to this hypothesis, one should expect that CRFs may eventually become prevalent in the entire region over time. The prevalence of CRFs, however, displays a great variation across neighboring regions in South America. While CRF12_BF (and related BF recombinants) attains a very high prevalence (>50%) in Argentina [12–14] and Uruguay [15], it displays an intermediate prevalence (<20%) in Chile and Paraguay [18, 19] and is almost completely absent in Brazil [11, 118–120]. Moreover, there is recent evidence that this CRF may be declining in prevalence in Argentina, through the analysis of vertically infected children [121]. Similarly, although the CRF28/29_BF and CRF31_BC variants reach a high prevalence in the cities of Santos (Sao Paulo state) [122] and Porto Alegre (Rio Grande do Sul state) in Brazil [106, 123], respectively, they are rarely found in other neighboring cities [120, 123, 124]. This indicates a fast, but geographically contained expansion of those CRFs, sometimes limited to a specific locality.

An alternative hypothesis suggests that difference in the rate of expansion of distinct HIV-1 clades in South America may reflect a variation in the efficiency of different transmission networks. According to this hypothesis, some HIV-1 clades spread faster, because they encounter a more favorable local transmission chain. It has been proposed that the prevalence of CRF12_BF is higher in South American countries whit more extensive IDU epidemics [16]. IDU populations are thought to represent extremely fast chains of virus transmission, and initial expansion of CRF12_BF through such efficient networks may explain its rapid initial growth rate in Argentina and Uruguay. Of note, a fast rate of dissemination (1.5 years^−1^) was reported for HIV-1B in a cohort of men having sex with men (MSM) in Italy [116], while variable growth rates (from 0.5 years^−1^ to 1.4 years^−1^) were described for HIV-1B spreading in a number of MSM transmission chains in the UK [114]. These evidences support the notion that the rate of expansion of HIV-1 in a given population is determined by the efficiency of the transmission network, rather than by the specific genetic composition of the viral strain.

The history of HIV epidemic in South America has been largely clarified by the application of bioinformatic tools developed from evolutionary genetics models of demography, population genetics, and phylogenetics. Over the last decades, novel algorithms have been implemented that improved parameter estimates and unveiled unique aspects of HIV spatial-temporal dynamics in the South American continent. However, unresolved issues still remain regarding uneven geographical and chronological sampling that warrant further assessment, which once overcome will greatly enhance the robustness of the estimates.

## Figures and Tables

**Table 1:** Coalescent estimates of epidemic growth rate of HIV-1 clades in South America.

Subtype	Demographic model	Molecular clock	Gene	Growth rate(year^−1^)	Reference
B	Exponential growth	Strict	*pol* (PR)	0.23 (0.20–0.26)	[105]
			*pol* (RT)	0.35 (0.31–0.40)	
			*pol* (PR/RT)	0.4 (0.2–0.7)	[106]
	Logistic growth	Strict	*env (C2-V3)*	0.54 (0.32–0.78)	[87]
			*pol (PR-RT)*	0.56 (0.35–0.80)	

F1	Logistic growth	Strict	*env (C2-V3)*	0.61 (0.40–0.86)	[87]
			*pol (PR-RT)*	0.59 (0.31–0.92)	

C	Exponential growth	Strict	*pol* (PR)	0.77 (0.62–0.93)	[105]
			*pol* (RT)	0.63 (0.51–0.75)	
			*pol* (PR/RT)	0.7 (0.3–1.0)	[106]
	Logistic growth	Strict	*env (C2-V3)*	0.77 (0.45–1.14)	[111]
			*pol (RT)*	0.70 (0.41–1.00)	
		Relaxed	*env (C2-V3)*	0.87 (0.50–1.29)	
			*pol (RT)*	0.81 (0.40–1.26)	

CRF12_BF	Logistic growth	Relaxed	*vpu*	2.24 (0.21–4.56)	[95]
		Strict	*pol (PR-RT)*	1.08 (0.79–1.44)	[96]
		Relaxed		1.22 (0.85–1.64)	

CRF28_BF/CRF29_BF	Logistic growth	Relaxed	*pol (PR-RT)*	1.18 (0.64–1.38)	[97]
			*gag*	1.20 (0.59–1.47)	

CRF31_BC	Logistic growth	Strict	*pol (RT)*	1.26 (0.61–2.10)	[111]
		Relaxed		1.27 (0.44–2.26)	

CRF38_BF	Logistic growth	Strict	*pol (PR-RT)*	0.83 (0.31–1.81)	[96]
		Relaxed		0.92 (0.41–1.75)	
